# From molecules to dynamic biological communities

**DOI:** 10.1007/s10539-013-9364-4

**Published:** 2013-02-05

**Authors:** Daniel McDonald, Yoshiki Vázquez-Baeza, William A. Walters, J. Gregory Caporaso, Rob Knight

**Affiliations:** 1 Department of Computer Science, University of Colorado at Boulder, Boulder, CO USA; 2 BioFrontiers Institute, University of Colorado at Boulder, Boulder, CO USA; 3 Department of Chemistry and Biochemistry, University of Colorado at Boulder, Boulder, CO USA; 4 Department of Molecular, Cellular and Developmental Biology, University of Colorado at Boulder, Boulder, CO USA; 5 Department of Biological Sciences, Northern Arizona University, Flagstaff, AZ USA; 6 Argonne National Laboratory, Institute for Genomics and Systems Biology, Argonne, IL USA; 7 Howard Hughes Medical Institute, Boulder, CO USA

**Keywords:** Microbiome, Timeseries, Microbial community analysis, Operational taxonomic units

## Abstract

Microbial ecology is flourishing, and in the process, is making contributions to how the ecology and biology of large organisms is understood. Ongoing advances in sequencing technology and computational methods have enabled the collection and analysis of vast amounts of molecular data from diverse biological communities. While early studies focused on cataloguing microbial biodiversity in environments ranging from simple marine ecosystems to complex soil ecologies, more recent research is concerned with community functions and their dynamics over time. Models and concepts from traditional ecology have been used to generate new insight into microbial communities, and novel system-level models developed to explain and predict microbial interactions. The process of moving from molecular inventories to functional understanding is complex and challenging, and never more so than when many thousands of dynamic interactions are the phenomena of interest. We outline the process of how epistemic transitions are made from producing catalogues of molecules to achieving functional and predictive insight, and show how those insights not only revolutionize what is known about biological systems but also about how to do biology itself. Examples will be drawn primarily from analyses of different human microbiota, which are the microbial consortia found in and on areas of the human body, and their associated microbiomes (the genes of those communities). Molecular knowledge of these microbiomes is transforming microbiological knowledge, as well as broader aspects of human biology, health and disease.

## Introduction: the revolution in DNA sequencing provides new insight into a range of microbial phenomena

Microbial ecology used to be a small and specialized field that struggled to identify more than a tiny proportion of the Earth’s microbial biodiversity. Part of the problem was due to the prevalence of pure-culture methods, in which microorganisms had to be removed from their natural environments (which included communities of other organisms) and cultured in laboratories. Recent advances in molecular techniques, sequencing technologies and computational methods have enabled researchers to explore the microbial world at unprecedented levels, with a focus on the natural habitats of microorganisms. The combination of these advances has so far produced remarkable insight into the role of microorganisms in human health and their powerful effects on the natural world, while at the same time developing novel evidence about the evolution and diversification of life on Earth. In this article, we discuss how these advances have allowed researchers to create new lines of inquiry, we summarize important biological and philosophical results from recent publications, and we discuss how our improved understanding of microbial ecology may affect our lives in the coming years.

The last decade has seen a transformation and democratization of DNA sequencing (Shendure and Ji 2008). High-throughput sequencing, of the type necessary to characterize the complex microbial communities that inhabit our bodies, used to be the exclusive province of a few large sequencing centers: only research groups with access to substantial resources could engage in sequencing projects. Now, a benchtop machine that fits in an individual investigator’s laboratory can produce billions of 100-nucleotide sequences per month. For comparison, a bacterial genome from the gut is typically about three million nucleotides and the human genome is about three billion nucleotides. However, the number of bacterial genomes that inhabit a human implies that they contribute far more genes than does our human genome (Turnbaugh et al. 2007). Playing music from a digital file once required a high-end workstation but can now be performed on a handheld device because transistors can now be packed more densely onto a microchip. In the same way, characterizing the types (e.g., the strains, species or phyla) of microbes present in a given sample (the microbiota) or the genes present in these microbes (the microbiome) are problems that can be addressed with a fixed amount of sequencing that is rapidly becoming cheaper and more accessible.

These transformations in sequencing technology have correspondingly changed what it means to undertake a sequencing project. When sequences were very expensive (in the late 1970s and early 1980s), it was a substantial accomplishment to sequence even one gene from one species. Correspondingly, the focus was on identifying genes that acted as the best phylogenetic markers. These were short fragments of sequences from which inferences about the patterns of evolution were likely to match the inferred patterns of evolution of the corresponding species. These markers therefore provided efficient readouts of evolutionary history while minimizing sequencing costs. For example, ribosomal RNA genes, which play essential structural and catalytic roles in the ribosome and are thought to be almost exclusively vertically transmitted (Lawrence 1999; Amann et al. 1995), have been especially useful for reconstructing phylogenetic trees, including phylogenetic trees of organisms that have not been isolated in pure culture (Pace 1997). Initial studies focused on the 5S rRNA gene (Woese and Fox 1977), although expansion to longer rRNA genes, notably the small subunit rRNA, has allowed substantially greater phylogenetic resolution (Lane et al. 1985; Winker and Woese 1991). Here we describe several conceptual changes deeper sequencing has led to already, and will refine in the future.

## From catalogs to robust, reproducible community patterns

The initial focus on cataloging the rRNA genes in individual species allowed phylogenies of known taxonomic groups to be reconstructed. This work provided the framework for our initial understanding that life on Earth falls into at least three distinct lineages: the Archaea, the Bacteria, and the Eukarya (initially described as the archaebacteria, the eubacteria, and the urkaryotes, respectively) (Woese and Fox 1977). These findings, which focused on sequencing DNA from known species, were soon complemented by a radical idea: that these phylogenetic marker genes could be isolated from *unknown* species via bulk DNA extraction directly from the environment. This technique, pioneered by the Pace lab (Pace et al. 1986), allowed researchers to start building catalogs of the known and unknown organisms, the DNA of which was present in any given environment. As the cost of sequencing DNA declined, the focus on sequencing single marker genes such as the 16S rRNA gene expanded to include shotgun metagenomic surveys, in which total DNA extracted from a sample is fragmented and sequenced. Both approaches are widely employed today. Marker-gene surveys are used to investigate the microbiota of a sample, and metagenomic surveys are used to investigate both the microbiota and the microbiome of a sample. These two views of microbial communities can yield different findings, because functional genes are frequently transferred horizontally (i.e., between different lineages). In contrast, rRNA genes are almost always transferred vertically. However, several recent studies have shown similar patterns emerging from studies involving both types of data (Turnbaugh et al. 2009a; Fierer et al. 2012b; Harris et al. 2013).

The 26 years of sequencing since Pace’s first community sequencing efforts have revealed a picture of 85+ phyla within the bacteria alone, and in some cases as many as 15 new candidate phyla have been detected in a single study (Ley et al. 2006; Harris et al. 2013). The bacterial and archaeal census has been estimated to reach as many as 10^6^–10^9^ species (Schloss and Handelsman 2004), when calculated using sequence similarity criteria. Robust patterns of microbial community composition have now been observed, in a wide range of host-associated and free-living contexts. For example, human body sites are highly distinct from one another and highly diverse among individuals (Costello et al. 2009; HMP-Consortium 2012). Although any two humans are >99 % identical in their genome composition (Venter et al. 2001), there are no species-level OTUs (operational taxonomic units) shared across the gut microbial communities of all humans (Yatsunenko et al. 2012). This lack of shared OTUs suggests that many of the phenotypic differences that we see between humans may arise from differences in our microbiota, rather than differences in our genomes. We suspect that this observation will drive many advances in medicine in the coming years. For example, lean and obese individuals differ systematically in their gut microbial communities (Ley et al. 2006; Turnbaugh et al. 2009a; Knights et al. 2011) but much less so in their genomic composition. Obesity can be identified 90 % of the time using the bacteria in the feces alone (Knights et al. 2011), but with only 58 % accuracy from variations in the genomes of different individuals (Sandholt et al. 2010). Similarly surprising insights have arisen in environmental microbiology. For example, pH has been found to be the main driver of microbial communities in soil (Lauber et al. 2009; Rousk et al. 2010; Chu et al. 2010; Fierer et al. 2012a), and salinity plays a crucial role in structuring both free-living bacterial and archaeal communities across many environments (Lozupone and Knight 2007; Caporaso et al. 2011b; Tamames et al. 2010; Auguet et al. 2010). These patterns can be striking: for example, seasonal patterns in marine water microbial diversity are highly reproducible in the Western English Channel (Gilbert et al. 2012), with the same organisms dominating microbial communities in different seasons annually. However, most of the organisms present in any given season are found even at just a single time-point if more sequences (millions rather than thousands) are collected from the sample (Caporaso et al. 2012). These results suggest that seasonal differences do not arise from the presence or absence of community members, but rather from variations in the abundance of organisms that are always present. This finding reinforces the point that much of what we think we know about the microbial world may be limited by the amount of sequencing that it is cost-effective to perform. The work to catalog Earth’s microbial diversity has thus produced a compendium of rich and detailed observations, and efforts such as the Earth Microbiome Project (Gilbert et al. 2010; Knight et al. 2012) will round out our encyclopedia of our microbial world. But cataloging alone is insufficient: a list of the species present in a rainforest, for example, speaks little to the interactions, functions or potential of the organisms so listed.

The problem with phylogenetic marker gene surveys, such as the 16S rRNA gene sequencing projects described above, is that they tell us the ‘who’, without the ‘how’, thus failing to answer the most pressing questions. For instance, how can an organism live at pH 0 (Edwards et al. 2000), and what can such capacities teach us about the potential for pollution mitigation or for life on other planets? Endeavors such as the Genomic Encyclopedia of Bacteria and Archaea (GEBA) (Wu et al. 2009) perform whole-genome sequencing on organisms that are as phylogenetically divergent as possible from previously sequenced organisms. Even a small amount of this phylogenetically targeted genome sequencing provides novel gene discovery that greatly outpaces gene discovery from organisms chosen arbitrarily or at random. Targeted sequencing can inform us about the reproducibility of the evolutionary process among organisms from different lineages that adapt to similar environments. For example, comparative genomics based on whole-genome data, and linked to rich evolutionary history and detailed environmental information (derived from marker gene databases and marker gene surveys, respectively), can offer insights into which types of biochemical or regulatory functions are necessary to survive in a given environment. These results enable an understanding of the systems biology of microbial communities, which can ultimately be applied to engineer microbial communities to treat disease, generate electricity, or clean up hazardous waste sites. However, marker gene surveys still improve our understanding of microbial ecology and enable novel findings and technological applications. We will focus on this technique for the remainder of the paper to show how this is the case.

## How do we know which microbes are present?

A key problem with studies of the microbiome lies in determining which organisms are present. All stages of the process, including DNA extraction, amplification of specific target genes, clustering of sequences, and identification of taxonomic group are prone to both error and bias (Hamady and Knight 2009). As the number of sequences involved in a given study has grown, reliance on advanced computational methods has increased (Gonzalez and Knight 2012). However, the algorithm that is chosen can have large impacts both on beliefs about what organisms are present in a given environment (Liu et al. 2008) and how many kinds of organisms are present (Kunin et al. 2010; Quince et al. 2009). Even defining *kinds of organisms* is complicated at the microbial level. In lieu of a robust definition of a microbial species (Cohan 2002), the percentage of sequence identity of a marker gene is often used to define operational taxonomic units or OTUs. For example, most 16S rRNA gene-based studies treat a cluster of sequence fragment ‘reads’ (the output of a DNA sequencing instrument, and thus the typical observation in studies of microbial communities) that are >97 % identical to one another as members of the same OTU. 97 % identity is treated as a proxy for species-level groupings of organisms, although this definition is known to be problematic for several reasons. One is that the rate of evolution of the 16S rRNA gene differs among taxonomic lineages, so the same number of differences in the sequence may represent different times since divergence from a most recent common ancestor. The choice of algorithm for assigning sequences to OTUs can also have a large impact on which sequences are clustered into the same OTU and on how many OTUs are observed in a study. For example, it is not clear whether a 97 % sequence identity threshold means that each sequence added to an OTU must be 97 % similar to all other sequences in the OTU cluster, or whether each sequence should be 97 % similar to the sequence that defines the center of the cluster (i.e. the cluster centroid) (Schloss and Handelsman 2005; Schloss and Westcott 2011). Because neither laboratory nor computational protocols are standardized, reported differences among studies often stem from differences in methodologies rather than from differences in the underlying biology. And because techniques for performing meta-analyses of microbiome data are still only emerging, it is often difficult to standardize a reanalysis, and comparisons of results across studies and especially among laboratories must be performed with caution.

**Fig. 1:**
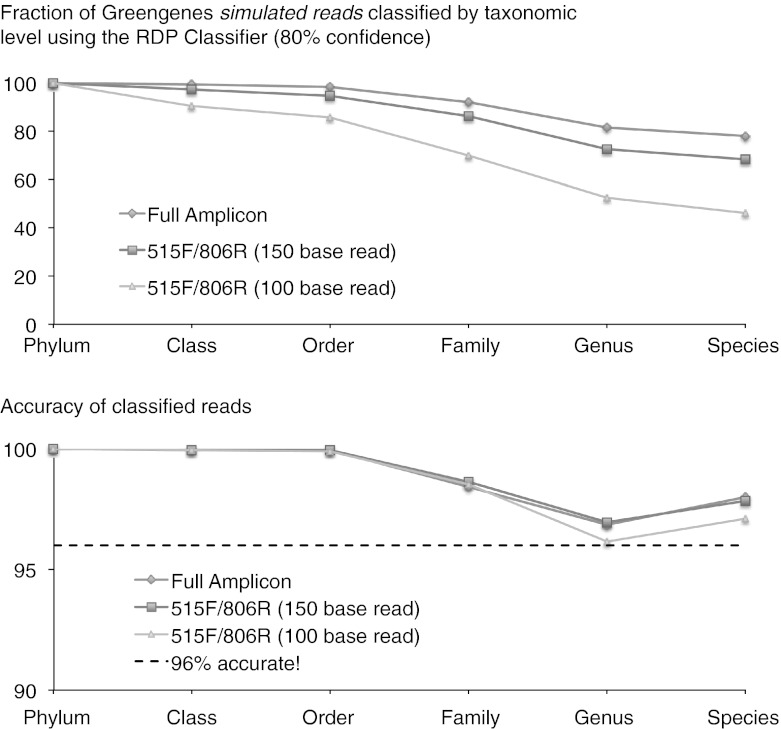
Relationship between sequencing read-length and our ability to classify sequences using the RDP Classifier, a popular taxonomic assignment method based on oligonucleotide frequencies (Wang et al. 2007). Simulated sequences were generated from 16S genes to represent the complete sequence between the 515F/806R primers (the “full amplicon”) or shorter 150 or 100 base pair reads from the 515f forward primer

Modern marker-gene-based studies often investigate the composition of microbial communities at the OTU level, due to difficulties in relating counts of short DNA sequence fragments to named species. Although short reads of sequences (100–400 bases is currently typical, depending on sequencing platform) from the genomes of well-studied organisms can often be assigned at least to the family level, and sometimes at the genus or species level, many sequences cannot confidently be assigned to known named taxonomic groups. The limitation here is primarily the amount of information present in short reads of marker genes for differentiating closely related taxa. Figure One shows that when working with the most informative region of the 16S rRNA gene for broad analyses of bacterial and archaeal communities, the fraction of reads that can be assigned to taxonomic groups increases as expected with the length of the sequence. In real-world experiments (as opposed to the simulation presented in Fig. 1) this effect is exacerbated by PCR and sequencing biases and errors.

Our inability to assign detailed taxonomy to short reads is often not important for many of the questions that are interesting to address at the community level. Phylogenetic diversity calculations allow us to determine the relative similarity of microbial communities, using similarity of the fragment of the marker gene as a proxy for the relatedness of the organisms represented by those marker genes. Although in principle horizontal gene transfer, the movement of genes among different genomes, could obscure the phylogenetic pattern, in practice the difference in gene content between two organisms closely tracks the differences in marker genes such as the 16S rRNA gene (Zaneveld et al. 2010; Konstantinidis and Tiedje 2005). However, there are cases in which genomes with identical 16S rRNA genes have markedly different properties (e.g., *Bacillus cereus,* a harmless soil bacterium, and *Bacillus anthracis*, the causative agent of anthrax, are almost indistinguishable except for a plasmid that confers pathogenicity (Ivanova et al. 2003)). Additionally, our conclusions are limited by our depth of sequencing (i.e., the number of marker gene sequence reads collected from a sample). A study that collects 1,000 sequences per sample will miss species that are only present at an abundance of one cell in a million. These limitations to knowledge are widely appreciated by specialists, but are often omitted in popular accounts and in descriptions for non-specialists.

## Is there a core human microbiome?

Our initial expectations of the microbial diversity living within and on human beings were limited and biased because relatively few microbes can be grown in culture (Rappé and Giovannoni 2003; Staley and Konopka 1985) and because many phylogenetically and functionally distinct kinds of microbes are difficult to distinguish by morphological or biochemical characteristics. For instance, *Escherichia coli* was believed to be a common and abundant gut microorganism inhabiting most members of the human population. However, culture-independent surveys based on 16S rRNA gene sequencing and/or shotgun metagenomic sequencing (in which all the DNA from a given community is extracted and analyzed) typically find it at less than 1 % abundance in the gut of healthy adults (Eckburg et al. 2005; Turnbaugh et al. 2009a; Costello et al. 2009; Qin et al. 2010). The scientific and medical community sought to determine the “core” microbiome of humans at the level of microbial species shared by everyone (Turnbaugh et al. 2007). Surprisingly, such a core does not seem to exist at the level of species; instead what appears to be shared are microbial functions (Turnbaugh et al. 2009a; Qin et al. 2010). One suggestion is that there might be a few types of common but only partially overlapping (or perhaps non-overlapping) microbial communities. One study found just three “enterotypes” or types of gut bacterial communities in human populations (Arumugam et al. 2011), although this simplistic picture appears not to be true when additional subjects and populations are considered (Wu et al. 2011; MacDonald et al. 2012; Jeffery et al. 2012; Claesson et al. 2012; Yatsunenko et al. 2012; HMP-Consortium 2012). However, the idea that human gut microbial communities might be classified into just a few types is conceptually appealing and has received much media attention (Brandon 2011; Yong 2012; Zimmer 2011), so debate on this topic is likely to continue. The microbial diversity revealed due to improvements in culture-independent techniques, in part due to the vast decrease in sequencing costs noted above, has been remarkable. There are no shared OTUs across the gut communities of all humans, even at a depth of coverage of one million sequences per sample (HMP-Consortium 2012). This unexpected finding has given rise to the idea of microbes as personal identification markers (Fierer et al. 2010). In addition, because monozygotic twins differ in their microbiota (Turnbaugh et al. 2009a; Yatsunenko et al. 2012), it could be argued that our microbiota are more personally unique than our own genomes.

In some sense, whether or not there is a core microbiome is a purely definitional issue. Finding a core depends on the level at which sequences are aggregated (grouping together more similar or more distantly related groups of organism, for example), the abundance threshold that may be set deliberately or may be intrinsically limited by technology or study design (for example, if only 1,000 sequences per sample are collected, organisms that are as rare as one in a million microbes will be missed), and the fraction of individuals that a taxon must appear into be considered “core” (for example, the MetaHIT consortium used a 50 % threshold (Qin et al. 2010)). Some kind of core can always be defined. A more productive research direction is to ask whether there are systematic differences among the microbial communities of every human that can be correlated with the physiological state of each individual.

## Microbial community states associated with disease

Much attention has focused on testing whether differences in microbial diversity correlate with physiological states, especially disease states. For example, Ruth Ley, Peter Turnbaugh and colleagues in the laboratory of Jeffrey Gordon embarked on an exploration of changes in the microbiota associated with obesity in different mouse models. This seminal work revealed robust differences in the gut communities of these mice compared with lean mice, both in the case of genetically induced obesity in the *ob/ob* leptin model (Ley et al. 2005) and in diet-induced obesity (Turnbaugh et al. 2008). Remarkably, increased adiposity was transmissible to genetically normal mice on a standard, calorie-controlled diet by transferring these microbial communities from the obese mice to the normal mice (Turnbaugh et al. 2006, 2008). The major taxonomic difference between the mice microbiota was the relative abundance of the phyla Bacteroidetes and Firmicutes. This finding has been shown to hold for human hosts as well (Ley et al. 2006), although the same pattern has not been replicated in all human studies (Duncan et al. 2008; Schwiertz et al. 2010). As mentioned above, we can now predict—based on the microbial community composition alone—whether an individual is obese or lean at 90 % accuracy (Knights et al. 2011) while predictions based on host genomic markers perform little better than chance (Sandholt et al. 2010). Interestingly, these predictions work best when the microbes are classified into broad groups. Clustering the sequences into groups at the 80 % sequence identity level (corresponding approximately to bacterial phyla) actually works better than clustering the sequences into groups at the 97 % sequence identity level (corresponding approximately to bacterial species) for classifying people as lean or obese. These more detailed analyses at the species-proxy level do, however, provide better resolution when classifying multiple samples from the same site (Knights et al. 2011). A possible explanation for the improved predictability using phylum-level classification could be that differences in biochemical pathways are differentially represented across phyla but conserved across OTUs within phyla. These biochemical pathways are the primary features that differentiate obese from lean individuals. Models trained on data that are too specific (i.e., clustered at 97 % identity rather than a lower percent identity) are prone to overfitting, and have reduced predictive capacity. But it is important to bear in mind that the phylogenetic levels at which bacteria are associated with particular states may vary considerably, depending on the ecology of the particular phenotype or disease.

Recent large-scale endeavors, such as the Human Microbiome Project (NIH 2012), the American Gut (Human-Food-Project 2012) and the Personal Genome Project (Personal-Genome-Project 2012) are opening up new opportunities for analysis because they are building a base of healthy microbiomic data against which disease states (collected by some of these projects) can be contrasted. This is important because of the breadth of diseases associated with the microbiome. Disease states that have been found to be associated with features of the microbiome include inflammatory bowel disease (Frank et al. 2007; Michail et al. 2012), wasting diseases (Gordon et al. 2012), obesity (Kallus and Brandt 2012), halitosis (Kazor et al. 2003), dental caries (Yang et al. 2012), and perhaps even autism (Finegold et al. 2010). The gut microbiome appears to be causal for certain disease states, and is not just a biomarker. Causality can be inferred when, for example, fecal transplantation (and thus microbiota inoculation) in human subjects is used successfully to treat inflammatory bowel disease (IBD—primarily ulcerative colitis) (Landy et al. 2011) and insulin sensitivity associated with metabolic syndrome (Vrieze et al. 2012). These results indicate that gut microbes play an active role in these disease states and are not merely effects of the host’s condition. It is possible that in the not-to-distant future a microbiome sample will become a normal component of a health checkup. Microbiome analyses may be used to diagnose disease and could provide possible avenues for the prevention of disease through predictive tests. As we mentioned above, molecular samples from microbial communities may track or predict disease states better than does the human genome.

## Changes in the microbiome over time

Microbial ecology shares similarities with traditional ecology, yet there are some important differences. In the ecology of macroorganisms, it is often possible to observe interactions directly, such as predation or competition for resources. Such observations are much more difficult in the microbial world, and ecological interactions must often be inferred from statistical variations in sequence data instead. Species definitions, although notoriously problematic even for macroorganisms, are even more difficult in microbes, and operational definitions based on similarities in DNA sequences must be used instead (as already discussed). Additionally, the cost of DNA sequencing posed a barrier until recently to collecting the detailed time-series and spatial datasets that are necessary for ecological modeling in microorganisms. However, some aspects of microbial ecology are substantially easier than in large-organism ecology. For example, the reliance on DNA sequence data means that with advances in technology, even a deep sampling of the population (millions of individuals) can be performed rapidly, and observation biases are likely to be less profound than when attempting to glimpse rare and elusive insects or mammals. The ability to collect large-scale information about microbial populations is likely to allow classical ecological models to be applied to the microbial world far more effectively than has been possible in macroecology, because more types of microbes can be simultaneously observed with large population sizes and with replicated sampling.

Ecological principles offer more than just ways to stratify the human population (e.g., by disease state). At infancy, our microbial populations go through remarkable changes in structure prior to reaching a resemblance to most adult communities. Inoculation is not necessarily from our mothers, and is substantially influenced by delivery mode. Microbial communities of children delivered vaginally initially tend to resemble their mother’s vaginal communities, while the microbial communities of children delivered by C-section initially tend to resemble human skin communities. Skin inoculations may be obtained from the mother, the medical staff involved in the delivery, or hospital surroundings (many of which harbor communities resembling human skin) (Biasucci et al. 2010; Dominguez-Bello et al. 2010). Stabilization of the microbiota of human children occurs around the third year of life (Yatsunenko et al. 2012), but routine disruptions, adjustments and fluctuations appear to be normal in healthy individuals (Costello et al. 2009; Caporaso et al. 2011a). While in general, the intra-individual microbiome variation is less than inter-individual, the amount of variability over long time periods (Caporaso et al. 2011a) gives rise to the idea of microbial “weather” in which microbial communities react to dietary and health conditions (even as they causally affect them). This phenomenon may be especially important in determining the health of the elderly (Claesson et al. 2012).

**Fig. 2:**
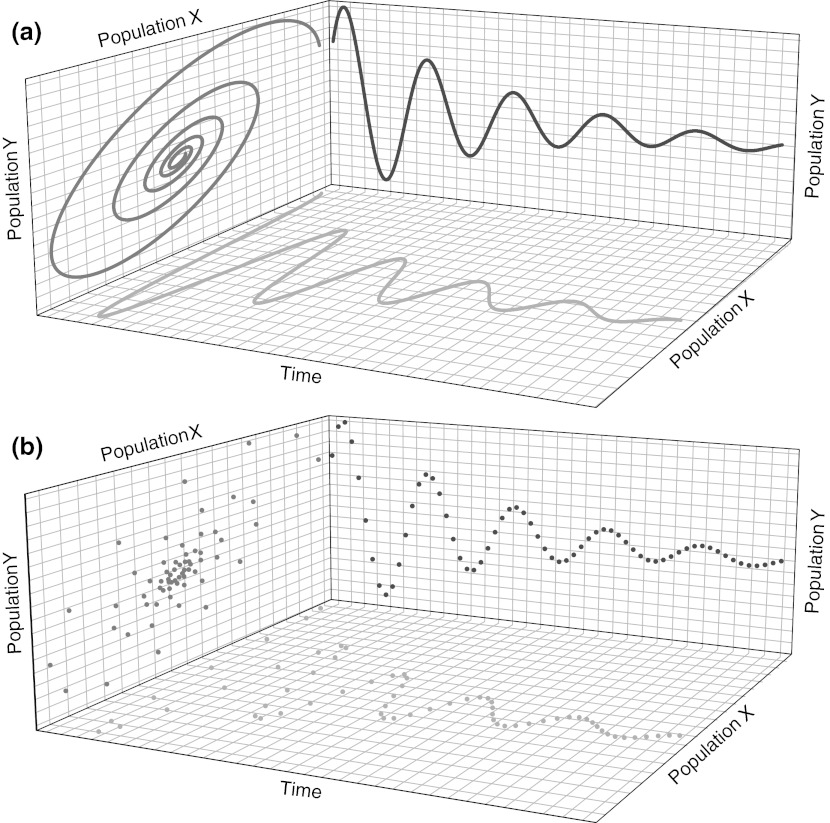
Predator-prey dynamics for two species X and Y lead to a scatterplot (relating sampled species abundances) that is interpretable when successive time-points are connected (**a**). If, however, the information about time were not included (**b**), these dynamics would appear uncorrelated because when X is high, Y can be either high or low, and vice versa. Thus, even in a completely deterministic system, it is impossible to tell whether two species interact with each another simply by examining multiple samples in which both are present. However, this technique is widely used in practice despite its limitations. Figure adapted from (Holling 1973)

A revelatory aspect to studies of the microbiome is that classical ecological models and datasets previously only obtainable for a few economically important systems, such as fisheries, are now testable on the microbial scale because of the ability to assess simultaneously the relative abundance of thousands of species in thousands of samples (Gonzalez et al. 2011). However, this move towards accounts of microbial communities in terms of alternative stable states and dynamical systems (Costello et al. 2012; Lozupone et al. 2012; Gajer et al. 2012) is not entirely without peril. In the absence of theories of underlying causes, defining the number and boundaries of these states can be technique-dependent and implicitly theory-laden in ways difficult to identify—especially by investigators who are not specialists in the relevant mathematical techniques. With the availability of larger datasets and the ability to track communities over time, key ecological concepts such as resilience, alternative stable states, predator–prey cycling, and bottom-up versus top-down regulation of ecosystems will be increasingly important. However, it is equally important not to forget the lessons learned from past applications of these techniques, especially in traditional ecological modeling. For example, it has been known for almost four decades that Lotka-Volterra predator–prey dynamics with time lags produce patterns that would appear as completely uncorrelated between two species that in fact do interact deterministically (Fig. 2) (Holling 1973). However, this fact is routinely ignored in network analyses that seek to find connections among organisms by building a network in which nodes correspond to organisms, and edges correspond to pairs of organisms that are correlated. Correlation is usually assessed by determining whether the abundances of two taxa are correlated across a set of samples, typically using the Pearson correlation coefficient that assumes that all interactions are linear. In other words, taxa are linked if their correlation coefficient exceeds an arbitrary researcher-defined threshold. These networks are often used to find groups of organisms that “co-occur”, presumably because of shared environmental preferences or because of mutualistic ecological interactions. Hence these network methods, which often rely on linear correlations among organisms to detect relationships (Qin et al. 2010; Steele et al. 2011; Barberán et al. 2012), would incorrectly assert organisms to lack ecological connections even when these connections are fully deterministic. This happens simply because the inference procedure requires an understanding of the time-evolution of the system in order to find these causal links.

The analysis of time-series in microbial ecology has also been limited because the performance of standard signal processing methods is degraded with uneven sampling periods and small numbers of data points (Moller-Levet et al. 2003; Mason 1978; Mallat 1989). Such degradations have historically been common in microbial ecology datasets due to the cost of obtaining the data. However, we have already obtained valuable information about the temporal dynamics of a few microbial communities, such as the assembly of an infant’s gut microbiome and its transition towards a healthy human adult gut microbiome (Koenig et al. 2011). In the few cases in which even sampling has been performed or can be assumed, techniques exist to detect abrupt disruptions (Beltran et al. 1994; Mallat and Zhong 1992). In these contexts, such disruptions could mean one of the interventions that has been shown to have large effects in mice or humans such as diet change (Turnbaugh et al. 2009b) or antibiotic administration (Dethlefsen et al. 2008; Dethlefsen and Relman 2011). Therefore, as in disease surveillance, choosing a specific analytical approach (for example co-occurrence analysis, clustering analysis, and control systems analysis) depends to a large extent on whether the goal is to monitor a trend, detect an outbreak or provide general awareness of the possibility of change (Robertson and Nelson 2010).

## Conclusions and outlook

Overall, the ability to collect far larger amounts of sequence data has led to much broader and deeper characterizations of the human microbiome and microbial communities in other habitats, especially when linked to rich contextual information about the provenance and status of each sample (Knight et al. 2012). In particular, the increased use of time-series studies (enabled by the decline in the cost of sequencing) allows us to apply for the first time a wide range of ecological models to the microbial world. Perturbation experiments are especially important for understanding how microbial communities change and for understanding groups of species that change together and interact in complex ways. However, this expanded body of ecological data introduces substantial epistemic issues, especially in regard to how data are interpreted via models and concepts. For example, the definition of OTUs at both the organism and the gene level (e.g. in the construction of “gene catalogs” (Qin et al. 2010)) is in many respects a return to phenetic methods, which have been criticized due to their lack of theoretical justification and their instability when more data are added (de Quieroz and Good 1997). The methodological principle of clustering sequences at some threshold before analysis is also not well grounded theoretically. One example would be if a single nucleotide change in the 16S rRNA gene of a single species distinguished exactly lean from obese humans, or co-varied perfectly with disease severity in IBD. Such findings would be of enormous importance yet would be missed completely by current techniques. Similarly, we know that because of factors such as horizontal gene transfer, gene- and taxon-level analysis will not map precisely on to each another, yet the data to perform such analysis and the theoretical framework for reconciling differences is at this point largely lacking.

Some of the solutions to these problems are being sought in large-scale projects such as the Earth Microbiome Project (Gilbert et al. 2010; Knight et al. 2012). These research consortia are working towards understand relationships among microbial processes across different systems and timescales. They will be especially important for identifying which theoretical constructs across different scales and levels of analysis are especially useful both for understanding and predicting microbial community responses. And as this article has made clear, the availability of large datasets and the development of new methods with which to analyze them have already produced dramatic changes in how the microbial world is understood, and its relationship to the rest of the biological world. As the many human microbiome studies discussed above show, microbial ecology—especially molecular microbial ecology, even at its relatively crude stage of development—is transforming how human biology itself is understood. This transformation, which we expect to occur not just in human biology but in traditional ecology and biology more broadly, will raise philosophical issues that require the attention of scientists and philosophers. We have indicated just some of these issues, dealing with the units of analysis and the causal powers associated with them, and how imperfect methods and models become more refined and effective in the process of inquiry. Philosophy of biology itself can learn a great deal from these recent and future developments in microbial ecology, as other papers in this special issue demonstrate.
